# Investigation of different caprock morphologies on CO_2_ leakage and solubility trapping mechanism

**DOI:** 10.1038/s41598-025-03416-7

**Published:** 2025-05-25

**Authors:** Pradeep Reddy Punnam, Venkata Sai Teja Tatavarthi, Vikranth Kumar Surasani

**Affiliations:** 1 Department of Chemical Engineering, Birla Institute of Technology and Science, Pilani-Hyderabad campus, Hyderabad, 500078 India; 2 Department of Earth Sciences, Indian Institute of Technology (IIT) Bombay, Powai, 400076 Mumbai, India

**Keywords:** CO_2_ geological sequestration, Reactive transport simulations, Solubility trapping, Caprock morphologies, CO_2_ leakage, Carbon capture and storage, Chemical engineering

## Abstract

A thorough understanding of subsurface formation zones is critical for safe and long-term storage using CO₂ geological sequestration (CGS). A major concern in CGS is the risk of CO₂ leakage due to plume migration through structural faults and cracks in the caprock. This research investigates the effects of caprock morphologies on CO₂ plume migration, solubility trapping, and leakage risks using multiphase multicomponent reactive transport simulations. Three synthetic domains with varying caprock morphologies are modelled, incorporating geological subsurface features. The study reveals that the presence of cracks significantly impacts CO₂ entrapment and leakage. In the current analysis outcomes, in comparison between the synthetic domain-1 and − 2, due to the presence of an anticline structure in synthetic domain-1 reduced sweeping efficiency by 25%, which in turn influenced solubility trapping, with leakage recorded 15% lower in synthetic domain-2 compared to domain-1. In contrast, synthetic domain-3, based on the Deccan traps stairsteps morphology, showed 30% less leakage despite injecting 21.6 Mt of CO₂, compared to the 18.9 Mt injected into domains 1 and 2. This is due to the enhanced CO₂ plume migration through stairstep traps in the lateral direction, which promoted primary trapping followed by solubility trapping. The findings highlight the importance of geological structures in determining CO₂ migration patterns, sweeping efficiency, and leakage risks, contributing to the optimization of CO₂ storage strategies. These insights are critical for addressing gaps in CGS implementation and ensuring the safe and efficient sequestration of CO₂ over the long term.

## Introduction

Carbon Capture and Storage (CCS) is a key technology used by both industrialized and developing countries to reduce anthropogenic CO_2_ emissions^1^. In CCS, CO_2_ is captured from major emission sources, such as thermal power plants, metal industries, and fertilizer plants, then transported to a geological injection site where it is injected into the subsurface, typically beneath impermeable caprock layers, in its supercritical state for efficient and economical sequestration^2,3^. Once injected, the CO_2_ tends to move upward due to buoyancy, where it is trapped beneath the caprock layer through structural trapping. Some CO_2_ also becomes confined in the porous subsurface formation through residual trapping and interacts with resident fluids to dissolve into the reservoir, increasing density and enhancing solubility trapping^3,4^. The dissolution of CO_2_ in connate water leads to a decrease in pH, which can trigger geochemical reactions, causing mineral trapping through the formation of stable secondary minerals, effectively sequestering the CO_2_ permanently^3–7^.

It is imperative to explore potential challenges in CGS, particularly regarding CO_2_ leakage, to guarantee the efficacy and sustainability of this method^8,9^. Leakage can arise from various factors, such as geological faults and cracks, wellbores, and seals confining the CO_2_, it can jeopardize and damage environmental, ecosystem and further it can impacts soil and water acidification^10,11^. Additional emissions resulting from leakage impact climate, economy, and mitigation strategies. Even a relatively low leakage rate, such as 0.1% per year, could translate into substantial additional emissions, emphasizing the urgency of addressing this concern^12^. Economic repercussions include potential losses in carbon credits and burdens on future generations to rectify the situation, hindering global deployment in the energy sector and potentially causing technological shifts. Human health risks arise, particularly in densely populated areas, where high CO_2_ concentrations may lead to suffocation hazards. Compounding these challenges is the risk of compromised storage site integrity, especially if leakage occurs through geological faults, diminishing the overall efficacy of geological sequestration^10–12^.

The analysis of CO_2_ leakage in carbon capture and storage (CCS) is of paramount importance for the success and credibility of this climate change mitigation strategy. Rigorous simulations and thorough examination of CO_2_ leakage are necessary to comprehend how it influences the trapping mechanisms, emphasizing the critical need to ensure that the stored carbon remains securely sequestered over the long term^10–12^. Mitigating climate change by reducing atmospheric CO_2_ through CCS can only be effective if leakage risks are well understood and addressed. Understanding and addressing CO_2_ leakage is crucial for ensuring the integrity and sustainability of CCS projects, highlighting the critical role that comprehensive leakage analysis and simulation play in advancing carbon capture and storage as a key technology for combating climate change^10–12^.

The majority of studies that focused on CO_2_ leakage have primarily examined the phase transformation of injected ScCO_2_, varied petrophysical properties of geological reservoirs, and the composition and properties of rocks. The petrophysical properties of the formation layer, in conjunction with the structural strength of the caprock, are crucial subsurface parameters required to alleviate and disperse the stress exerted on the caprock. Numerous researchers have employed and formulated various indicators to facilitate risk assessment analysis^12–14^.

The monitoring and assessment of CO₂ leakage in subsurface geological formations has been a significant focus in the context of carbon capture and storage (CCS), as effective CO₂ storage is critical to mitigating climate change. Studies have employed various methods to track and predict CO₂ plume migration and leakage risks. Fawad and Mondol^15^ propose a combination of seismic and electromagnetic (EM) surveys to monitor CO₂ migration, focusing on detecting potential leakage pathways and improving storage security through time-lapse data on plume behaviour. This geophysical approach highlights the importance of accurate tracking in identifying areas where CO₂ might migrate out of storage zones, thus ensuring the integrity of storage sites^15^.

Alcalde et al.^10^, take a probabilistic modeling approach to evaluate CO₂ storage security, estimating leakage rates over millennia. Their findings emphasize that well-regulated CO₂ storage can retain more than 98% of injected CO₂ over 10,000 years. In comparison, poorly managed sites might retain over 78%, underlining the role of regulatory oversight in minimizing leakage. This study adds depth to our understanding of long-term CO₂ behavior and the variability of leakage risks based on site characteristics^10^.

Miocic et al.^16^, provide a geological case study on faulted sites, examining natural leakage over 420,000 years in Arizona, USA. They observed leakage rates to be considerably below 0.01% annually, demonstrating that, even in faulted reservoirs, geological storage can be a reliable mitigation technique if site selection is rigorous. Their findings highlight the influence of geological features on leakage risks and the potential of faults to act as both conduits and barriers depending on site conditions^16^.

The interaction between CO₂ leakage and shallow aquifers, particularly in carbonate environments, presents distinct challenges. Segura et al.^17^, investigate how CO₂-rich water percolates through porous limestone, noting that high CO₂ concentrations, salinity, and flow rates can significantly alter the petrophysical properties of aquifers. Initial permeability changes and pore-clogging dynamics offer insights into how CO₂-induced dissolution and fine particle transport could affect aquifer quality. This highlights the need to understand flow dynamics in predicting CO₂ leakage impacts on freshwater resources^17^.

Little and Jackson^18^, Xiao et al.^19^, and Zheng et al.^20^, collectively provide a detailed assessment of the chemical impacts and potential risks posed by CO₂ leakage on shallow groundwater systems, especially in carbonate aquifers. Little and Jackson^18^ conducted a 300-day laboratory incubation study simulating CO₂ infiltration in freshwater aquifers, where they observed a pH drop of 1–2 units that resulted in a marked increase in concentrations of metals such as manganese, cobalt, nickel, iron, uranium, and barium. Their findings identify manganese, iron, calcium, and pH as reliable geochemical markers of CO₂ leakage, highlighting the importance of aquifer buffering capacity, redox conditions, and metal mobility in shaping water quality outcomes. This study emphasizes the need to select deep geologic sequestration sites with minimal potential for adverse effects on groundwater^18^.

Complementing these findings, Xiao et al.^19^, performed a combination of column experiments and reactive transport simulations focused on the Ogallala aquifer. They observed that CO₂ leakage causes significant dissolution of carbonate minerals, particularly calcite and dolomite, releasing trace metals. Although most metal levels returned to baseline, manganese and uranium concentrations exhibited prolonged increases, indicating long-term contamination risks. This study’s reactive transport modeling highlights the need to consider geochemical reactions and transport dynamics when assessing CO₂ leakage impacts on shallow groundwater^19^.

Expanding on these findings, Zheng et al.^20^, provide a comprehensive review of CO₂ leakage effects on groundwater quality, highlighting potential increases in acidity, trace metal release, organic compound mobilization, and shifts in microbial activity. They identify lead (Pb), arsenic (As), and uranium (U) as primary contaminants of concern, often exceeding regulatory limits in affected areas, with carbonate aquifers particularly vulnerable to Pb and As contamination. The authors also propose an eight-step risk assessment procedure involving laboratory testing, modeling, and field evaluations to evaluate aquifer vulnerability and establish a systematic approach for groundwater quality management at carbon sequestration sites^20^.

Druhan et al.^21^, explore the reactive transport simulation of a CO₂ leak into an overlying aquifer, focusing on the use of caprock and hydrodynamic control strategies to mitigate leakage. By injecting a silica-bearing sealant, the study demonstrates the feasibility of reducing permeability and CO₂ flux, offering a potential remediation strategy for leaks through targeted injection. Their work highlights the potential for caprock formations to act as barriers and the value of hydrodynamic controls in active leakage scenarios, supporting the application of reactive transport modeling in designing geochemical mitigation approaches^21^.

Md Jamilur Rahman et al.^14^, conducted research on the caprock seal integrity. The study was focused on the geomechanical properties of shale caprock. The field-scale analysis was carried out on the Early Jurassic Cook and Johansen formation’s overlying Drake formation shales located offshore of Norway at the Horda Platform area. The field-scale investigation was carried out using fifty exploration wells and 3D seismic and 2D seismic lines. CO_2_ leakage and risk assessment were analyzed at the Aurora injection site of the Drake formation. It was observed that shale caprock diffused the stress induced during CO_2_ injection. In the risk assessment, the top seal of the formation was effective in reducing CO_2_ leakage. The author also recommended that numerical simulations are required to properly assess risk factors with various macroscale parametric variations^14^.

Together, these studies illustrate the complexities and risks of CO₂ leakage from geological storage sites and emphasize the need for comprehensive monitoring, regulatory oversight, and mitigation strategies to ensure the long-term security of CO₂ sequestration as a climate mitigation strategy. The presence of geological features like anticline and syncline can influence the entrapment and migration of CO_2_; during this movement of CO_2_, the plume may encounter cracks and faults, which further leads to CO_2_leakage. Afanasyev, Andrey et al.^13^, conducted research on the CO_2_ migration in the sloping aquifer in the un-dip direction. This analysis is carried out by generating governing equations using immiscible flow equations. The outcome from the empirical equation is validated with the numerical simulations. The relationship established for dip angle, porosity, permeability and saturation function was analyzed. Then, the outcome of this study was used for leakage analysis, risk assessment and uncertainty analysis^13^.

The type of caprock plays a critical role in determining the long-term stability of CO_2_ storage sites, with variations in mineral composition, geomechanical properties, and chemical reactivity directly influencing containment efficiency. An effective caprock usually needs to have higher capillary pressure than the pressure exerted by CO_2_, or it can cause leakage^22^. The most effective mineral composition of caprock is fine-grained siliciclastic, gypsum, and anhydrite. Caprock properties like porosity and permeability would alter due to interaction between CO_2_-brine-rock^23^. Previous experimental work on the interaction of CO_2_-shale in batch reactor indicated that CO_2_ could dissolve shale at a low kinetic rate. Crushed samples of shale rock collected from Pottsville Formation, USA, with three crushed samples (A: Clay 75% Quartz 25%, B: 65% Clay and 35% Quartz, C 50% Clay and 50% quartz) are used for experimentation each has a different ratio of clay quartz and feldspar and are reacted with CO_2_ in batch reactor, it was observed that initially the pH climbed to 8.6 for few days and later the buffering capacity (alkaline effect) of shale reduced and gradually decrease in pH is observed reflecting the acidic nature of CO_2_. BET technique is used to obtain pore geometric properties like surface area and sample C surface area reduced reflecting mineral precipitation^24^.

CO_2_ high-pressure injection can weaken the caprock, and when stress exceeds the material’s resistance, microcracks can form, leading to CO_2_ leakage^25–27^. Globally, 22 caprock leakage incidents were linked to integrity issues, mainly due to poor geological study and excessive injectivity pressure, as seen in the In Salah project in Algeria, which stopped in 2011^28–30^. Caprock’s mineral composition, particularly quartz, is crucial in assessing its integrity with CO_2_ reactions that can increase permeability and cause leakage. XRD analysis shows quartz dominates the caprock, making it less reactive with CO_2_. Triaxial tests on caprock samples found peak strengths between 48 MPa and 80.5 MPa, classifying them as strong rocks that can handle injectivity stress with quasi-elastic behaviour making them ideal for CO_2_ storage^31^.

In the initial implementation of CCS, the viability of the establishment and reservoir structural integrity assessment are crucial for any subsurface geological formations. A detailed risk assessment and associated parametric analysis should be evaluated to ensure the successful execution of CCS technology. Risk assessment is the key to accepting and implementing CCS in any region for any formation type. The numerical simulation analysis is vital for risk assessment to analyze the CO_2_ migration in the subsurface formation domain to predict the structural integrity along with the leakage probability in the domain. One of the significant drawbacks in the numerical simulation is the lack of geological subsurface data available for simulations; the subsurface formation is a complex structure to simulate the influences of various reservoir parameters on the CO_2_ migration and entrapment to assess the structural integrity and safety of CCS^12^.

In the current study, the initial two numerically modelled synthetic geological domains considered were partially adapted from prior research^5^. The previous study focused on caprock morphology, demonstrating its influence on primary and solubility trapping mechanisms. The presence of geological features like anticline structures has influenced the migration pathway and shown that caprock morphology affects sweeping efficiency^3,5,6^. The sweeping efficiency has been observed to influence solubility trapping: when sweeping is high, more of the CO_2_ plume that spreads will be in contact with the connate water, increasing solubility trapping^3^. In the synthetic domain with geological features, the CO_2_ plume stagnated safely, but solubility trapping was somewhat low. Regardless, CO_2_ stored within specific geological features would reduce monitoring costs^3,4^. Petrophysical properties, including porosity and permeability, play an equally vital role in CO_2_ migration and trapping^3,6^. Formations with lower porosity and permeability create more resistance to CO_2_ flow, which can enhance residual trapping by immobilizing CO_2_ in smaller pore spaces. Conversely, higher permeability may allow CO_2_ to migrate faster, reducing the efficiency of both structural and residual trapping^3,6^. The petrophysical characteristics also affect the sweeping efficiency, as formations with lower permeability are more likely to trap CO_2_^3,6^. Additionally, structural- and residual trapping mechanisms are influenced by the proximity of the injection site to high-quality formation traps, which can increase CO_2_ storage capacity. The rate at which CO_2_ is injected further determines migration patterns and storage effectiveness, with higher injection rates potentially reducing trapping efficiency due to faster CO_2_ migration^5,6^.

Based on prior research findings and published literature, it is well-established that the morphology of the upper subsurface caprock significantly influences the trajectory and migration patterns of CO_2_ plumes^3,4,6^. It plays a pivotal role in determining the extent of CO_2_ entrapment within the geological domain. The presence of cracks near anticline features can affect CO_2_ entrapment, making the careful selection of subsurface CO_2_ injection points crucial for effective carbon capture and storage (CCS) technology^3,4,6^. Additionally, factors like pressure, which is affected by the petrophysical properties of the formation, influence solubility trapping. High reservoir pressures, resulting from lower-permeability formations, can increase the dissolution of CO_2_ into formation water, thus enhancing long-term CO_2_ storage^3,4,6^. However, maintaining caprock integrity is vital to prevent CO_2_ leakage and ensure that CO_2_ remains trapped within the formation.

While previous studies have explored caprock integrity in CO₂ geological sequestration (CGS), there is a knowledge gap regarding how varying caprock morphologies affect CO₂ migration, leakage risks, and trapping mechanisms. Most research has focused on caprock permeability and fracture propagation, often simplifying caprock structures. In contrast, real-world formations exhibit complex morphologies like anticlines, flat surfaces, and stair-stepped features, which can alter CO₂ plume behavior. Anticlines may concentrate CO₂ plumes beneath structural highs, increasing leakage risks, while flat or irregular caprocks promote lateral spreading and enhance solubility trapping. This study addresses this gap by examining the impact of different caprock geometries on CO₂ plume migration, solubility trapping efficiency, and leakage predictability using multiphase multicomponent reactive transport simulations across three synthetic domains. The research links morphological variations to key storage metrics, offering insights for optimizing CGS site selection and injection strategies.

To effectively mitigate the implications of CO_2_ leakage, a comprehensive strategy involving detailed analysis, experimental studies, and rigorous simulations is imperative. It is crucial to recognize the significant challenges posed by large-scale experimental studies, such as the need for extensive infrastructure and substantial financial investments. In light of these challenges, simulations emerge as a practical and valuable alternative. By leveraging advanced simulation techniques, researchers can conduct in-depth analyses, model various scenarios, and predict outcomes related to CO_2_ leakage. These simulations not only provide cost-effective solutions but also offer valuable insights into the risks associated with CO_2_ leakage, aiding in the development of robust mitigation strategies. Integrating both experimental data and simulation-based analyses ensures a well-informed approach to address the multifaceted implications of CO_2_ leakage, striking a balance between scientific rigour and practical feasibility.

The objective of this ongoing research is to examine the impact of caprock leakage on CO_2_ entrapment and investigate the role of caprock morphology on CO_2_ sequestration. For this purpose, three synthetic geological domains with distinct caprock morphologies were considered to explore the influence of geological structures and features on entrapment and structural integrity. The migration and sweeping efficiency of CO_2_ were analyzed by studying the distribution of CO_2_ mole fraction and pH within the domain. The structural integrity and leakage analysis investigation also involved monitoring the CO_2_ mole fraction and evaluating solubility trapping for entrapment. The structural integrity assessment was conducted by examining reservoir pressure distribution within the domain. The numerical techniques employed in this research are explained in the theory section, while the modelling of synthetic domains is detailed in the geometrical modelling of computation domains. The results section comprehensively explains the impacts of caprock morphology on CO_2_ migration, sweeping efficiency, CO_2_ leakage, and CO_2_ entrapment. This is followed by conclusion section on the current research.

## Theory

This simulation analysis was carried out utilizing multiphase flow and reactive transport models. In the CO_2_ sequestration analysis, reactive transport models are used to investigate geochemical interactions in both geospatial and temporal dimensions. To explore the interactions between the water and CO_2_ plume, the multiphase and multicomponent mass and energy balance equations were utilized and investigated^32,33^. In this simulation analysis, the mineral trapping reactions are neglected to reduce the complexity and condense the required computational time. The mass balance equation depicted below consists of three terms. The first term refers to the accumulation within the control volume, while the second term relates to the net flow. The third term, represented as *Q*_*w*_ for water and *Q*_*c*_ for carbon dioxide, accounts for the source and sink components of the system. It encompasses the injection and removal of species from the domain, respectively. For liquid (*l*) and gaseous (*g*) states, the mole fractions of water (*w*) and carbon dioxide (*c*) are denoted by $\:{X}_{w}^{l\:or\:g}$, and $\:{X}_{c}^{l\:or\:g}$. Porosity is represented by the symbol *Ø*. Saturation for different phases is represented by *S*_*l or g*_, densities are denoted by *ρ*_*l or g*_, and molecular diffusion (D) is represented by *D*_*l or g*_.

1 $$\:\frac{\partial\:}{\partial\:t}\left[{\varnothing}\left({S}_{l}{\rho\:}_{l}{X}_{w}^{l}+{S}_{g}{\rho\:}_{g}{X}_{w}^{g}\right)\right]+\nabla\:\left[{q}_{l}{\rho\:}_{l}{X}_{w}^{l}+{q}_{g}{\rho\:}_{g}{X}_{w}^{g}-{\varnothing}\left({S}_{l}{{D}_{l}\rho\:}_{l}{\nabla\:X}_{w}^{l}+{S}_{g}{{D}_{g}\rho\:}_{g}{\nabla\:X}_{w}^{g}\right)\right]={Q}_{w}$$

2 $$\:\frac{\partial\:}{\partial\:t}\left[{\varnothing}\left({S}_{l}{\rho\:}_{l}{X}_{c}^{l}+{S}_{g}{\rho\:}_{g}{X}_{c}^{g}\right)\right]+\nabla\:\left[{q}_{l}{\rho\:}_{l}{X}_{c}^{l}+{q}_{g}{\rho\:}_{g}{X}_{c}^{g}-{\varnothing}\left({S}_{l}{{D}_{l}\rho\:}_{l}{\nabla\:X}_{c}^{l}+{S}_{g}{{D}_{g}\rho\:}_{g}{\nabla\:X}_{c}^{g}\right)\right]={Q}_{c}$$

3 $$\:{q}_{p}=\frac{-k{k}_{rp}}{{\mu\:}_{p}}\nabla\:\left({P}_{p}-{\rho\:}_{p}gz\right)$$

In the subsequent explanation of this manuscript, the subscript *p* symbolizes phases, whereas the letters *l* and *g* represent the liquid and gas phases. The parameter terms pressure, relative permeability, fluid viscosity, and mass density for a phase *p* are denoted by the notations *P*_*p*_, *k*_*rp*_, *µ*_*p*_ and *ρ*_*p*,_ respectively. The terms *k*,* g*, and *z* indicate the absolute permeability, the acceleration due to gravity, and the vertical height. To solve the conservation equations, this simulation study uses auxiliary equations for phase saturation, mole fraction, and capillary pressure, as illustrated in Eq. 4. 5 and 6.

4 $$\:{S}_{l}+{S}_{g}=1$$

5 $$\:{x}_{g}+{x}_{l}=1$$

6 $$\:{P}_{C}={P}_{nw}-{P}_{w}$$

In the current simulation analysis, the relationship between capillary pressure and saturation is considered from the Brook Corey function; this relationship between effective saturation, *S*_*e*_, and capillary pressure, *P*_*c*_, is illustrated in Eq. 7. The Brooks Corey Burdine’s relation is used for the relationship between saturation and relative permeability. The relationship between the relative permeability of the liquid phase, *k*_*rl*_, and the gases phase, *k*_*rg*_, with effective saturation of different phases are illustrated in Eq. 8 and Eq. 9. Brooks-Corey and Burdine equations appear in the literature in a variety of forms. For this simulation investigation, the saturation-capillary pressure and saturation-relative permeability relations have been obtained from PFLOTRAN^34,35^. Both of these relations have been utilized in the current simulation analysis.

7 $$\:{S}_{e}={\left(\alpha\:{P}_{c}\right)}^{-\lambda\:}$$

8 $$\:{k}_{rl}=({{S}_{l}}^{eff}{)}^{3+2/\lambda\:}$$

9 $$\:{k}_{rg}={\left(1-{S}_{g}^{eff}\right)}^{2}\left[1-{\left({S}_{g}^{eff}\right)}^{3+2/\lambda\:}\right]$$

The effective saturation for the different phases can be estimated from Eq. 10 and Eq. 11, and the terms liquid saturation, residual liquid saturation, and residual gas saturation are represented by $\:{S}_{l}$, $\:{{S}_{l}}^{r}$ and $\:{{S}_{g}}^{r}$. The symbolic terms α and λ are empirical constants used in Eq. 10 and Eq. 11.

10 $$\:{{S}_{l}}^{eff}=\frac{{S}_{l}-{{S}_{l}}^{r}}{1-{{S}_{l}}^{r}}$$

11 $$\:{{S}_{g}}^{eff}=\frac{{S}_{l}-{{S}_{l}}^{r}}{1-{{S}_{l}}^{r}-{{S}_{g}}^{r}}$$

Equation 12 illustrate the multicomponent reactive transport modelling, which includes the reaction part in the numerical simulations. The terms $\varPsi$, *ϖ*, *υ*_*jm*_ and *I* denotes the concentration of the species, flux, stoichiometric coefficients and dynamic rate of reaction. The subscripts *j* and *m* represents primary species and minerals in the subsequent manuscript text.

12 $$\:\frac{\partial\:}{\partial\:t}\left[{\varnothing}\left({S}_{l}{\psi\:}_{j}^{l}+{S}_{g}{\psi\:}_{j}^{g}\right)\right]+\nabla\:\left({\varpi\:}_{j}^{l}+{\varpi\:}_{j}^{g}\right)=-\sum\:_{m}{\upsilon\:}_{jm}{{\rm\:I}}_{m}$$

Equation 13 illustrates the concentration of primary species, *j*, at phase *p*. The $\:{C}_{i}^{p}$ and $\:{C}_{j}^{p}$ are secondary species concentrations and primary species concentrations for phase *p*. The Secondary species reaction coefficient is represented by $\:{\nu\:}_{ji}$.

13 $$\:{\psi\:}_{j}^{p}={\delta\:}_{l}^{p}{C}_{j}^{p}+{\sum\:}_{i}{\nu\:}_{ji}{C}_{i}^{p}$$

Net flux, $\:{\varpi\:}_{j}^{p}$ is illustrated in Eq. 14, for primary species *j* for phase *p*. τ represents the tortuosity, and Dp represents the phase diffusivity coefficient.

14 $$\:{\varpi\:}_{j}^{p}=\left(-\tau\:\phi\:{S}_{p}{D}_{p}+{q}_{p}\right){\psi\:}_{j}^{p}$$

The *I*_*m*_ term represents the dynamic rate of reaction; the equation is illustrated in Eq. 15. Variables like the kinetic rate constant of a mineral, the surface area of the minerals, activity of the *i*^*th*^ species, and ion activity product are denoted by *K*_*m*_, $\:{A}_{m}\varnothing\:$, *a*_*i*_ and *Q*_*m*_.

15 $$\:{I}_{m}=-{K}_{m}{A}_{m}\varphi\:\left[{\prod\:}_{i}{a}_{i}^{ni}\right](1-{K}_{m}{Q}_{m})$$

The ion activity product equation is illustrated in the following Eq. 16; the term activity coefficients is denoted by $\:{\gamma\:}_{j}^{p}$, the subscript *j* represents secondary species concentration, and superscript *p* represents the phase.

16 $$\:{Q}_{m}=\prod\:_{j}{\left({\gamma\:}_{j}^{p}{C}_{j}^{p}\right)}^{{\upsilon\:}_{jm}}$$

The following Eq. 17 denotes the energy balance equation for the current simulation analysis. The porous rock density and heat capacity is denoted by *ρ*_*r*_ and *C*_*r*_. The source or sink term is denoted by *Q*_*e*_. Thermal conductivity, internal energy, enthalpy and temperature are denoted by $\:\kappa\:$, U, H and T.

In the current study analysis, the PFLOTRAN, open-source software, was considered due to its advanced capabilities in simulating complex multiphase, multicomponent, and multiscale reactive flow in porous media, particularly for CO_2_ geological sequestration. Its ability to handle unstructured morphology models and the ability to run efficient parallelization made it the optimal choice for this study. In this particular piece of research, our focus is primarily limited to the kinetics of CO_2_ dissolving and the following change in pH to track the solubility trapping process that is taking place in the computation domain.

## Modelling of the synthetic simulation domain

The numerical simulation model in this study is a theoretical framework designed to investigate fundamental caprock morphology effects on CO₂ leakage and solubility trapping. The synthetic geological domain was conceptualized based on structural analogues to the Deccan Traps, a large igneous province with basaltic formations relevant to CO₂ storage. However, due to the limited availability of high-resolution subsurface data from specific field sites, the model parameters (e.g., permeability, porosity) were derived from generalized ranges literature.

While the study focuses on theoretical scenarios, the findings provide critical insights for practical CO₂ storage projects. The results highlight how irregular caprock surfaces (e.g., undulations) influence CO₂ plume migration and dissolution rates. These findings can guide site selection by prioritizing formations with morphologies that enhance solubility trapping. The simulated dissolution patterns inform optimal sensor placement for leakage detection in field deployments.

**Fig. 1:**
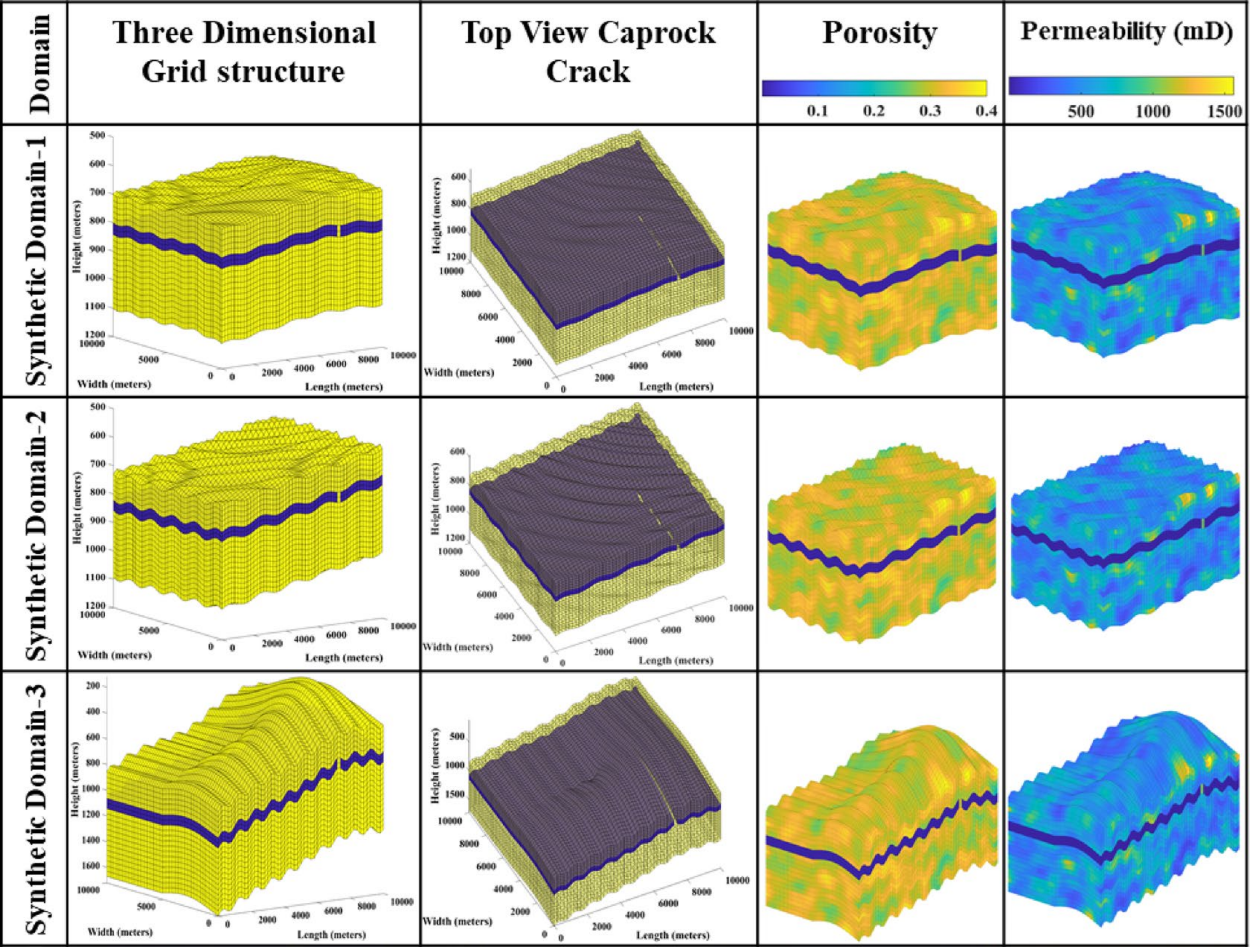
The figure illustrates a schematic diagram of the three-dimensional grid structure, the domain’s top view, and the distribution of petrophysical properties in three considered synthetic domains. In column three, the three-dimensional domain is made transparent so that the crack on the caprock can be viewed from the top view.

In this numerical analysis, three synthetic domains are modelled. The initial two synthetic domains considered in the current research analysis are partially adapted from the previous study^4^. The difference between the previously considered domains in the preceding study and the current synthetic domain is that the domain is incorporated with the faulty caprock in which the line fault is embedded, and it is assumed that once the CO_2_ plume encounters this fault, the CO_2_ plume tends to escape or leak from the targeted formation. As illustrated in Fig. 1, synthetic domain-1 has the anticline structure integrated into the synthetic computational domain, whereas, in synthetic domain 2, no geological feature is integrated except perturbation and sloping. The synthetic domain-3 is modelled based on the available data of the Deccan traps^5,36^. The stairsteps morphology is adopted from the available geological data^3,5,36^. The 50,000 grid cells are used for the initial two synthetic domains for the modelling, where 50 divisions are considered on the sides, and 20 divisions are considered in the depth direction (50 × 50 × 20) of the numerical domain. The physical dimension of synthetic domain-1 and 2 is 10 km ×10 km × 350 m. The synthetic domain-1 and − 2 injection points are nearly at the same location. For the synthetic domain-3, the physical dimension is 10 km ×10 km × 800 m. The synthetic domain-3 contains the sloping anticline domain with stairsteps traps embedded in it; similar geological features can be seen in the Deccan traps^3,5,36^. Table 1 provides information on the top, bottom, caprock layer thickness, and grid cell discretization of all three synthetic domains.

**Table 1:** Illustrate the grid cell discretization and thickness of the top, bottom and caprock layers.

Domains	Grid cells	Top section thickness	Caprock thickness	Bottom section thickness
Synthetic domain 1	50 × 50 × 20	87.5 m	35 m	227.5 m
Synthetic domain 2	50 × 50 × 20	87.5 m	35 m	227.5 m
Synthetic domain 3	50 × 50 × 20	200 m	80 m	520 m

The second column of Fig. 1 illustrates the three-dimensional grid structure of all three synthetic domains. It can be observed that the synthetic domain-1 and 2 start from approximately 600 m in depth, and the caprock is set around 800 m. In synthetic domain-3, the domains start from 200 m; similarly to synthetic domain-1 and − 2, the caprock is situated at 800 m. The synthetic domain-3 has a sloping feature that roughly resembles the Deccan volcanic province’s Deccan traps. The third column illustrates the top view of the transparent grid structure of the domain by highlighting the caprock and crack integrated into the domain. The fourth and fifth columns show synthetic domains’ porosity and permeability distribution. The porosity and permeability values are arbitrarily assigned. The petrophysical properties for all the synthetic domains are considered the same range; the porosity varies from 0.2 to 0.4, and the permeability ranges from 10 to 1500 mD, whereas for the caprock, very low porosity and permeability to act like a caprock rock layer or impermeable layer^3,5^.

The synthetic domain-1 and − 2 are considered in this research analysis to study the influence of geological morphology on CO_2_ migration. In the prior research, the modelled synthetic domains were considered to analyze the influence of migration and sweeping efficiency of CO_2_ plumes without the fault caprock embedded into the domain^3^. In the current simulation analysis, the presence of a fault near the anticline dome can keep the CCS project in a precarious position. To simulate a geological fracture, a line crack type is purposefully introduced into the synthetic domain and caused to travel through the anticline dome. The observed results have encouraged us to conduct a similar study on the available geological data domain. The synthetic domain-3 is modelled based on available geological data in its stairsteps and anticline dome structure. The stairsteps features are commonly seen in the Deccan traps, and to integrate the un-dip nature, the anticline dome was incorporated in the Synthetic domain-3. From this study, the influences of some morphological parameters can be analyzed and studied to a certain degree.

In the present simulation analysis, several assumptions were made to simplify the simulation framework and focus on the bulk movement and migration of CO₂ plumes across different morphological domains. It is assumed that the reservoir fluid is water without impurities, thereby eliminating the complexities associated with brine chemistry. Additionally, mineral trapping is not considered in the simulations, which implies that the petrophysical properties of the rock, such as porosity and permeability, remain static over geological time. This assumption allows for a more focused examination of solubility trapping and structural trapping mechanisms without the influence of geochemical reactions.

The simulations are conducted under hydrostatic conditions, where the fluid density varies with height according to *ρgh*, where *ρ* represents the fluid density, *g* is the gravitational acceleration (9.8 m/s²), and *h* is the reservoir depth. Dirichlet boundary conditions are applied to all models, ensuring fixed pressure and temperature values at specified boundaries. The initial reservoir pressure is set at 70 bar, and the temperature is initialized at 75 °C across all domains. These parameters are consistent with typical subsurface conditions for CO₂ sequestration.

As the simulations are performed at a macroscopic scale, microscale and mesoscale phenomena are not explicitly resolved but may be indirectly reflected in the results. The primary aim of the simulations is to analyze the bulk movement and migration of CO₂ plumes across synthetic domains with varying caprock morphologies. This approach enables a systematic investigation of how geological features such as anticlines, flat surfaces, and stairstep structures influence plume dynamics, solubility trapping efficiency, and leakage risks over geological timescales.

## Results

### Base case: influences of caprock morphology on CO_2_ migration and leakage

In the current simulation analysis, the average initial reservoir pressure and initial reservoir temperature were considered as 77 bar and 75 °C for all three considered simulations. The injection rate considered for the synthetic domain-1 and − 2 is 0.63 Mt/year. The injection was carried out into the domains for the initial 30 years, and the remaining 2970 simulation years were reserved for post-injection observations. For the synthetic domain-3, the injection rate was fixed at 0.36 Mt/year for 60 years, and the remaining simulation time of 2940 years was reserved for post-injection analysis. The total injection amount of ScCO_2_ into the synthetic domain-1 and − 2 are about 18.9 Mt, and for the synthetic domain-3, it is about 21.6 Mt. In the simulations, the density of water is assumed to be 975.86 kg/m^3^, while the density of CO_2_ is considered to be 686.54 kg/m^3^. The viscosity of water is regarded as 0.3086 × 10^−3^ Pa.s, whereas the viscosity of CO_2_ is assumed to be 0.0566 × 10^−3^ Pa.s. The density of the rock is considered to be 2700 kg/m^3^, and the fluid diffusion coefficient in the reservoir is assumed to be 1 × 10^−9^ m^2^/s.

**Fig. 2:**
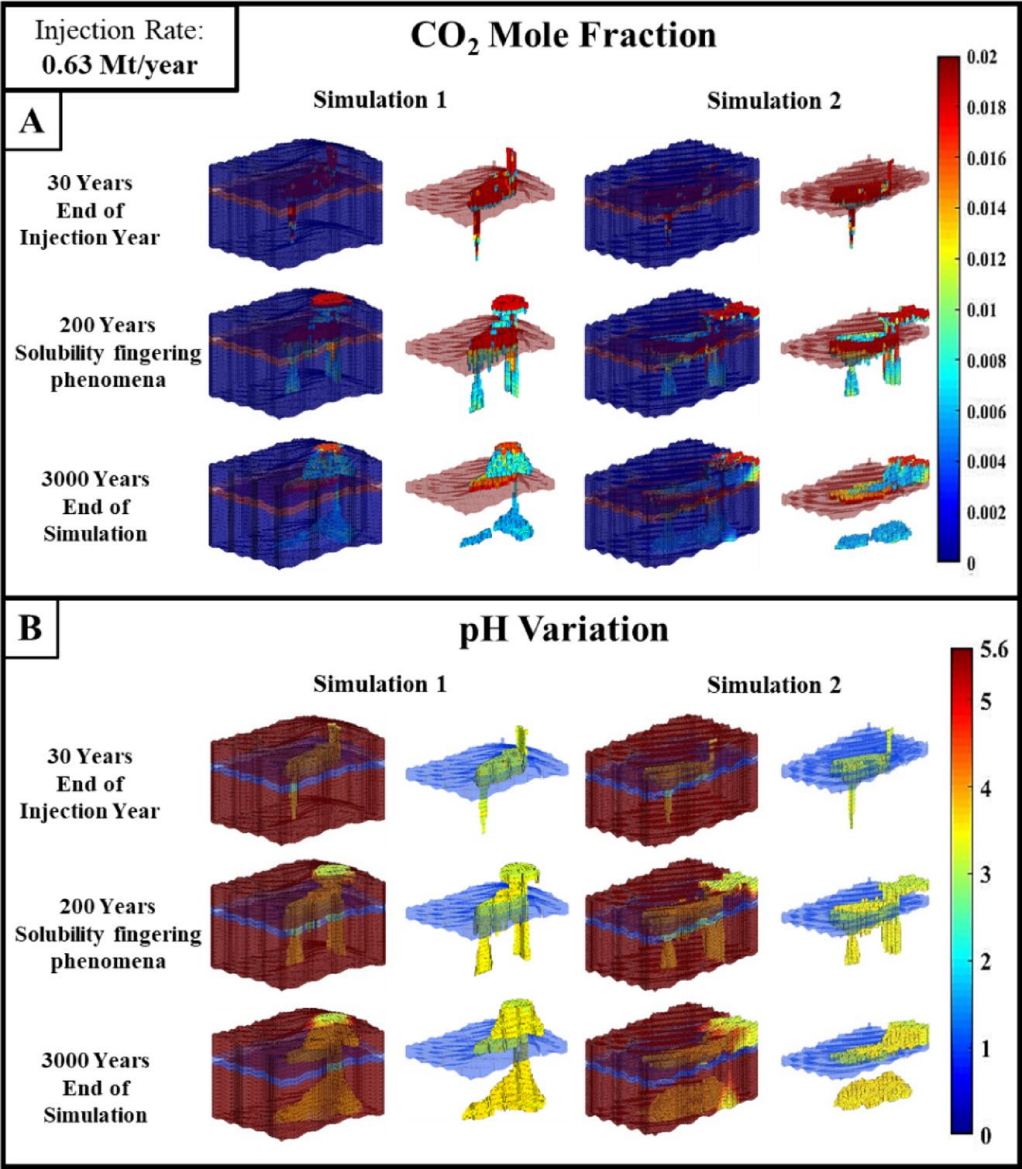
The figure illustrates (**A**) CO_2_ Mole Fraction and (**B**) pH variation over geological time scale during CO_2_ geological sequestration for synthetic domains 1 and 2.

The initial two synthetic domain simulation outcomes are illustrated in Fig. 2, along with their simulation results. The injection point, injection rate, and all other simulation parameters are the same in both simulations. The only difference between the synthetic domains is the difference in top caprock morphology. The cracks on the caprocks of both domains are situated at the same location. This simulation analysis investigates the way morphology influences CO_2_ plume propagation, sweeping efficiency, and CO_2_ leakage. In simulation 1, the synthetic domain-1 is used as a computational domain, whereas in simulation 2, the synthetic domain-2 is used. During the post-injection period, in both simulations, it was observed that the CO_2_ plume moved towards the elevated region. In simulation 1, due to the presence of an anticline dome in the synthetic modelled domain, the most amount of plume is seen moving in a narrow pathway and stagnating in the anticline dome. While in simulation 2, the synthetic domain-2 does not contain any geological features, the injected CO_2_ has spread under the caprock. In simulation 1, the synthetic domain’s crack is placed near the anticline feature to analyze the impact on CO_2_ leakage. From Fig. 2, by comparing two sets of simulations, it can be observed that the CO_2_ leakage is high in Simulation 1 compared to Simulation 2. In simulation 2, due to the high sweeping of the CO_2_ plume in all directions, only a small quantity of CO_2_ reached the crack. However, due to the CO_2_ stagnation in the anticline in the simulation 1 domain, more CO_2_ has been leaked than in the simulation 2 domain. This provided an intuition about the drawback of having cracks and leakage faults near geological features with elevation like anticline domes. The leakage can also be maximum if the crack is present in the narrow pathway in which the CO_2_ plume is migrating.

Figure 2A and B illustrate that a leaky caprock is incorporated into the domains, and the CO_2_ leakage can be visualized from the CO_2_ mole fraction and pH variation results. A line crack type is induced into the synthetic domain passing through the anticline dome, which acts as a systematic geological fracture. From the CO_2_ liquid mole fraction results at the 30 th year, it can be observed that after CO_2_ migrates into the bottom caprock anticline dome, it quickly escapes to the top caprock due to the presence of a crack on the caprock. The top caprock is crack-free and will restrict the injected CO_2_ from escaping to the Earth’s surface. Parameters like reservoir pressure, sweeping efficiency and solubility entrapment are analyzed to study the structural integrity of the domain during CO_2_ sequestration in the presence of leaky caprock. For instance, the solubility fingering phenomena at the bottom caprock was observed around the 200 th year, whereas at the top caprock, the solubility fingering was noticed around the 1000 th year, see Fig. 1. This may be due to the height of the CO_2_ plume stagnating in the dome and reservoir pressure distribution with respect to reservoir depth. The solubility trapping near the leaky caprock is crucial because it may lead to mineral precipitation, which might reduce the intensity of caprock leakage.

Compared to synthetic domain-1, synthetic domain-2 has a more significant portion of the domain covered by low pH in the bottom section of the domain, as illustrated in Fig. 2B. Comparing synthetic domains 1 and 2, synthetic domain 1 has a smaller volume covered and a lesser displacement efficiency, as seen in the bottom section of the domain. This is because the CO_2_ plume’s migration path was narrow as it got closer to the anticline’s centre. In the bottom section of the synthetic domain-2, the injected CO_2_ plume has an enhanced spreading in the lateral direction under the caprock, which increases the CO_2_ dissolution because due to the lack of anticline dome in synthetic domain-2, the contact area between the CO_2_ plume and resident water of reservoir was higher. It also contributes to the fingering phenomenon, which improves the vertical spreading in the depth axis. The low pH region created during the solubility trapping phase may result in a mineral reaction for the mineral trapping mechanism if the pH range of the domain is within acceptable limitations. In the synthetic domain-1, after CO_2_ leaked to the top section, the CO_2_ plume stagnated in the top section’s anticline dome. After some time, it can be seen that solubility trapping and low pH region dominated in the area, which might be an advantage if the mineral reaction near the crack of the bottom section caprock can contribute to crack healing.

**Fig. 3:**
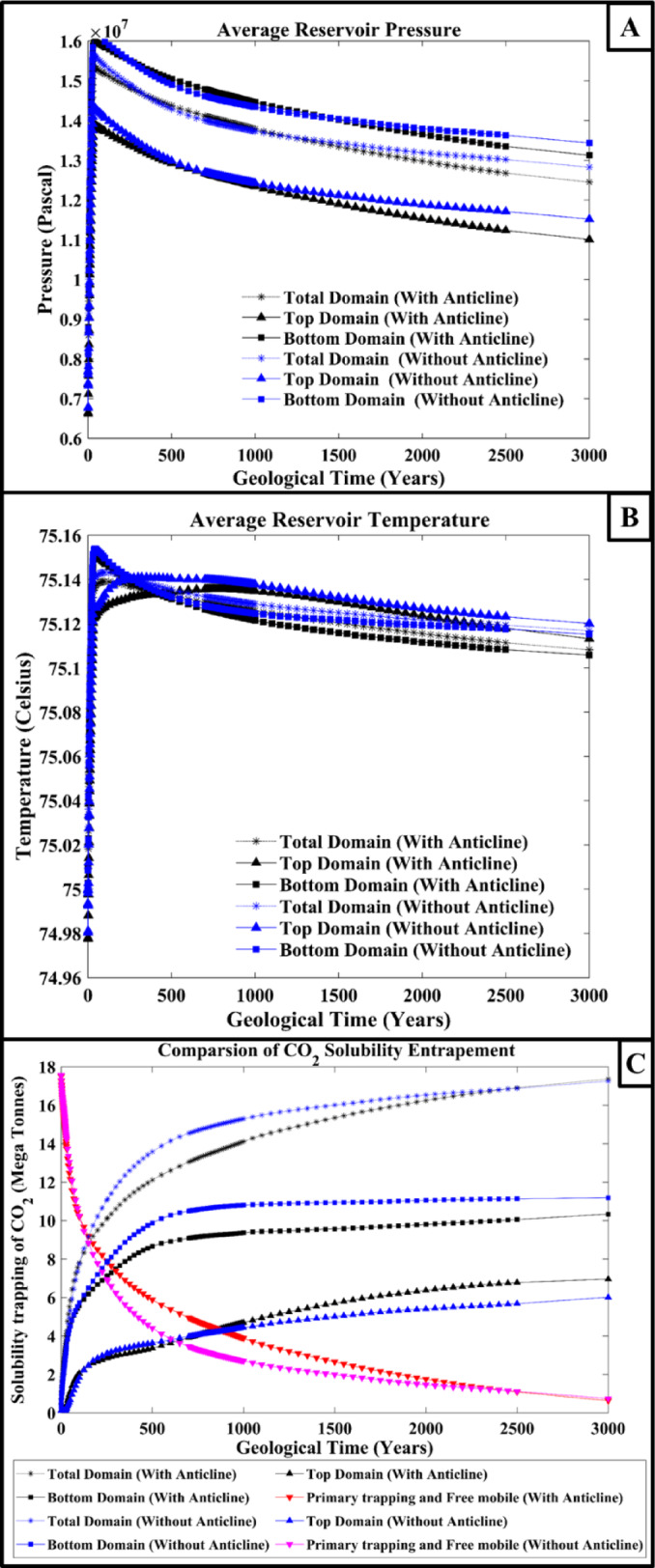
The figures show the results of the influence of caprock morphology on (**A**) Average reservoir pressure, (**B**) Average reservoir temperature, and (**C**) Solubility trapping of CO_2_ variation over a geological time scale for synthetic domain 1 and 2.

Figure 3 illustrates the average reservoir pressure, temperature and solubility entrapment percentage over a geological time scale. The pressure variation in the average reservoir pressure results is similar in both synthetic domains; see Fig. 3A. But, at the end of the simulation time, the average pressure in simulation 1 for the synthetic domain-1 decreased slightly. This might be due to the presence of a crack in the anticline dome. If there is no crack in the vicinity of the anticline dome, the pressure would be similar to or higher than the synthetic domain-2. Since the flow of the CO_2_ plume will be constrained in a narrow pathway, in the end, the CO_2_ will accumulate in the dome’s centre. This confined CO_2_ plume would increase the pressure at that section, but the pressure will get released in case of a crack presence. Compared to simulation 2, in simulation 1, a high amount of CO_2_ solubility entrapment was recorded in the top section of the domain because more CO_2_ escaped to the top section (see Fig. 3C). In simulation 2, in the absence of geological features, the large quantity of CO_2_ was spread in all directions; due to this, only a small quantity of CO_2_ reached the crack, and a small amount of CO_2_ leaked into the top section of the domain. The temperature variation was minimal and considered nearly negligible in the domain, see Fig. 3B.

### CO_2_ leakage in the stairsteps domain

**Fig. 4:**
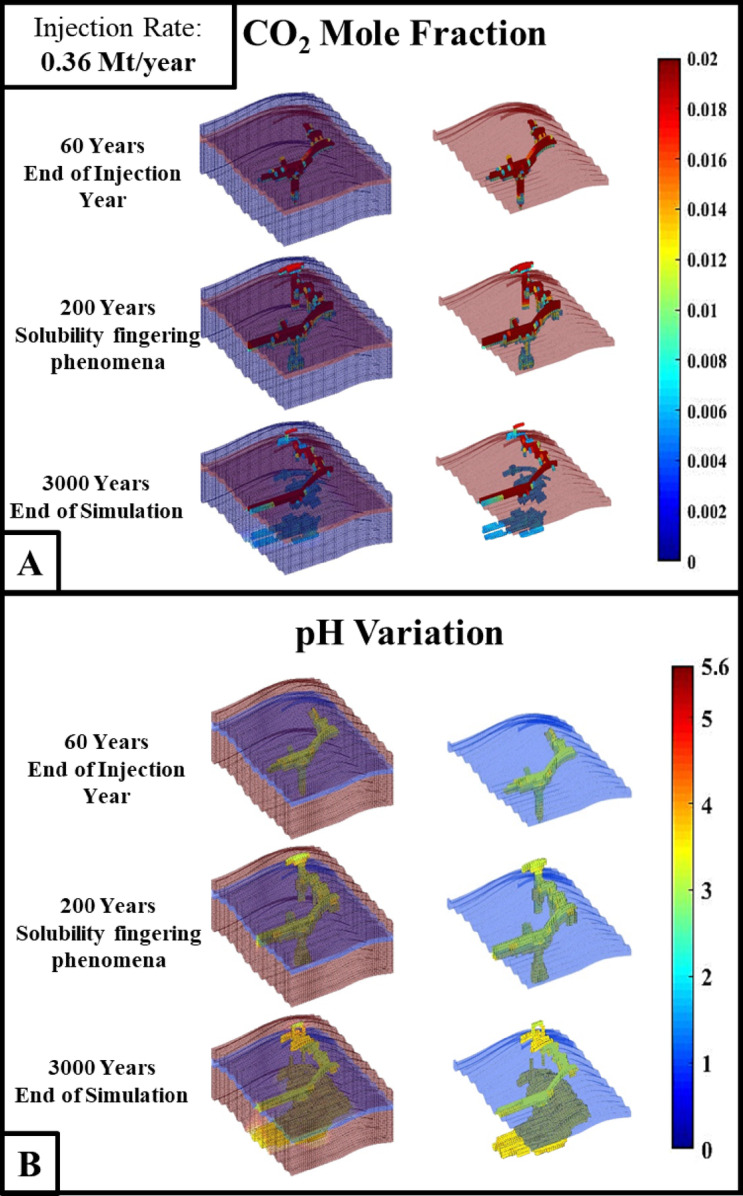
The figure illustrates (**A**) CO_2_ Mole Fraction and (**B**) pH variation over a geological time scale during CO_2_ geological sequestration for the synthetic domain 3 simulation.

This section carried out the CCS leakage analysis in the synthetic domain-3. Conducting CCS leakage analysis in this synthetic domain-3 will give an idea about plume migration, solubility trapping, and leakage predictability in the stairstep domain. The synthetic domain-3 is embedded with the anticline structure, stairstep features and sloping inclination. From Fig. 4A and B, it is observed that once the ScCO_2_ is injected into the domain, due to the influence of the stairsteps feature, the CO_2_ tends to spread in a lateral direction; when the laterally migrating CO_2_ plume comes under the influence of anticline structure, the plume tends to move upwards toward the high elevated region. The crack is located on the anticline structure, and it can be observed that the CO_2_ plume is escaping from that crack.

Figure 4B illustrates the pH variation in the geological subsurface. The low pH regions (pH 4–5) observed near the injection site correlate with peak CO₂ dissolution rates. These zones act as solubility ‘hotspots,’ where density-driven convection sustains fresh brine contact with free-phase CO₂. It can be seen that in the migration pathway, the pH of the domain was low, which indicates the solubility trapping in the migration pathway. The large volume of the bottom section of the domain is covered with low pH variation. From Figs. 2A and B and 4A and B, it can be observed that the solubility trapping was rapid in the bottom section compared to the top section. It might be due to the pressure distribution in the synthetic modelled domain. It is known that the pressure of the reservoir increases with the depth of the reservoir, so at a higher depth, the solubility trapping will happen at a higher pace.

**Fig. 5:**
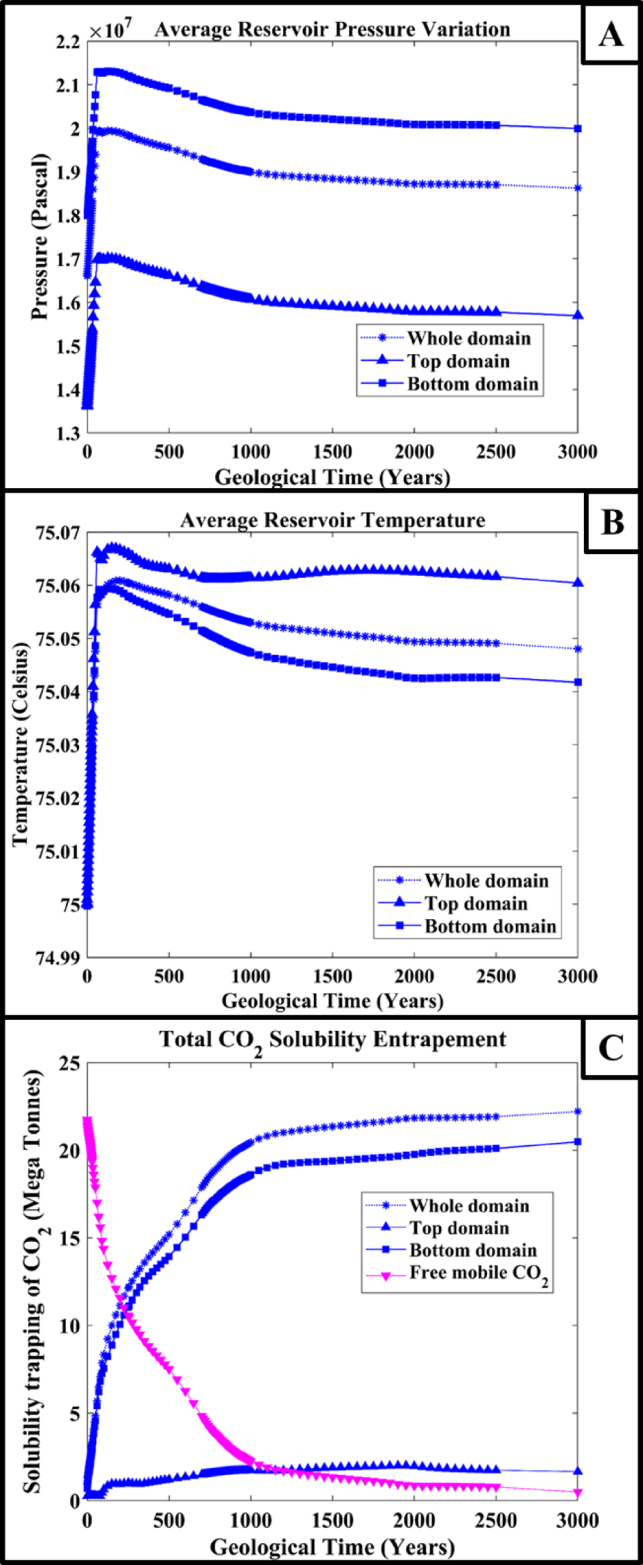
The figure shows the influence of caprock morphology on (**A**) Average reservoir pressure, (**B**) Average reservoir temperature, and (**C**) Solubility trapping of CO_2_ variation over geological time scale for the synthetic domain 3 simulation.

From Fig. 5A, the average reservoir pressure for the top section, bottom section and whole domains is illustrated. The pressure in the reservoir increases as depth increases; similarly, it is observed in the illustrated results of average reservoir pressure. The top section’s average reservoir pressure is low compared to the bottom section’s average reservoir pressure, and the whole domain average reservoir pressure recorded is in between the top and bottom section’s pressure variation. The injection was carried out around 1100 m below the Earth’s surface, which is why the higher in the average reservoir pressure is seen compared to simulation-1 and simulation-2 results. The temperature has not shown any significant variations in the domain; see Fig. 5B.

The solubility trapping recorded over a geological scale is illustrated in Fig. 5C. From this observation, it can be analyzed that solubility trapping is higher in the bottom section than in the top section. This might be because, as seen in Fig. 4A, most of the CO_2_ plume spread in the lateral direction and underwent solubility trapping. The reservoir pressure at the bottom section was high, which increased the solubility entrapment in the bottom section. While comparing the previous section’s results of synthetic domain-1 and − 2, it can be observed from the solubility trapping results that the top section of the domain solubility was high in synthetic domain-1 and − 2 compared to synthetic domain-3 with respect to the total injection quantity. This analysis also gives an idea of the selection of injection location with respect to the crack location, taking into consideration geological features and sloping nature to analyze the CO_2_ plume migration in the domain. The CO_2_ plume migration is one of the major parameters that should be investigated in order to study the structural integrity and CO_2_ leakage from the injected formation domain.

### CO₂ mass flux analysis

The study aims to analyze the CO₂ mass flux for all three scenarios. The CO₂ mass flux was calculated based on CO_2_ leaked into the top section of the domain, which is measured as the mass flux in kilograms per year to provide more granular insights into the amount of CO₂ escaping into the top section of the domain at various time intervals. The injection of CO₂ in the initial two synthetic domains was same constant for both cases, but the leakage dynamics were influenced by the presence and absence of the anticline structure. For the Synthetic domain-3 scenario, the injection was carried out for the first 60 years at an injection rate of 0.36 Mt/year, whereas in the first two synthetic domains was 0.63 Mt/year.

The CO₂ mass flux for each scenario was calculated by determining the difference in CO₂ amounts at successive time points (t_i+1_ and t_i_) in the top section of the domain, divided by the time interval (t_i+1_ - t_i_). This provided the rate of change in CO₂ leakage, which was subsequently presented in kilograms for a clearer understanding of the amount of CO₂ escaping to the surface. The calculation method used in this study adheres to standard mass flux calculations, ensuring that the temporal dynamics and leakage rates are accurately captured. The following Table 2 provides a detailed breakdown of the CO₂ mass flux for three case scenario**s**:

**Table 2:** Table of CO₂ mass flux for all scenarios.

Time (Year)	Synthetic domain-1 (kg/year)	Synthetic domain-2 (kg/year)	Deccan-Morphology (kg/year)
30	32,088	23,374	12,000
60	16,237	21,940	13,500
100	2,931	2,884	3,000
1000	2,034	1,151	2,100
1500	1,331	797	1,100
2000	583	600	900
3000	583	600	850

The CO₂ mass flux data provides a comparative view of how the CO₂ escapes into the top section of the domain under different geological scenarios. In the synthetic domain-1 scenario, the CO₂ leakage is initially higher, reaching 32,088 kg/year at year 30, due to the confined nature of the anticline structure. The presence of the anticline forces the CO₂ to move towards the dome and leak through fractures, resulting in higher leakage rates, particularly in the initial stages of CO_2_ injection.

In the synthetic domain-2 scenario, the CO₂ spreads more evenly across the subsurface, with a more gradual and consistent increase in leakage over time. The initial flux is lower at 23,374 kg/year at year 30, and it increases at a slower rate compared to the synthetic domain-1 scenario. This is due to the absence of a confined structure like the anticline, which allows the CO₂ to diffuse more evenly in all lateral directions, and only some quantity of CO_2_ plume encountered fault.

The Synthetic domain-3 scenario, on the other hand, shows a unique pattern due to the stairstep-like topography of the region. The CO₂ in this scenario is primarily forced to spread laterally, following the natural channels created by the uneven morphology. At year 30, the mass flux in the Synthetic domain-3 scenario is 12,000 kg/year, which is significantly lower than in the synthetic domain-1 scenario and synthetic domain-2 scenario during injection phase and at the end of the injection period at year 60, the CO2 mass flux recorded as 13,500 kg/year significantly less than the synthetic domain − 1 and-2. The lateral movement of CO₂ in this scenario, caused by the topographical features, results in a moderate and more consistent leakage rate over time.

## Conclusion

The current simulation analysis aimed to investigate how caprock morphology impacts CO_2_ leakage from a leaky caprock and its subsequent effect on CO_2_ entrapment, especially through solubility trapping, in the context of carbon capture and storage (CCS). The presence of a crack in the CO_2_ migration pathway has significant implications for entrapment percentage, structural integrity, and safe storage, all critical factors for the secure implementation of CCS. This study demonstrates the influence of geological structures and blended features of geological morphology on the movement of a CO_2_ plume in the presence of a crack, elucidating their impact on the plume’s entrapment percentage and structural integrity.

In this analysis, three synthetic geological domains with different caprock morphologies are considered to study the influence of geological structures and features on CO_2_ entrapment and structural integrity. It is observed that in comparison between synthetic domain-1 and − 2, the presence of anticline structure in the domain has decreased the sweeping efficiency of the injected CO_2_ plume by approximately 25%, which further influences the solubility trapping. Due to the high lateral sweeping in synthetic domain-2 compared to synthetic domain-1, a significantly smaller amount of CO_2_ plume reached the fault in synthetic domain-2, and the leakage recorded was 15% less. This study would also demonstrate the significance of geological features; if the crack was not located near the vicinity of the anticline dome, the synthetic dome could be a safer option for sequestration.

The study conducted on the synthetic domain-3, which was approximately modelled based on the available geological data of Deccan traps morphology, gives the intuition about the CO_2_ plume migration and influences of geological features like an anticline with an implication of CO_2_ leakage due to the presence of a crack near the anticline structure. During the initial years, the movement of CO_2_ was influenced by the stairsteps structure, and the CO_2_ plume mainly spread horizontally in a single stripe of the trap. When the CO_2_ plume comes under the influence of the anticline dome elevation, the CO_2_ plume tends to move in the up-dip direction, passing through stairsteps traps. It demonstrates that the geological structure has a dominant influence on the migration of the CO_2_ plume in the subsurface geological domain. While comparing the CO_2_ leakage into the top section of a domain in all the synthetic modelled domains, it can be observed that in synthetic domain-1 and − 2, which received 18.9 Mt of CO_2_, the leakage of CO_2_ into the top section was maximum when compared to synthetic domain-3, which despite receiving a larger injection volume of 21.6 Mt, showed approximately 30% less leakage. Because the CO_2_ plume migrated and percolated through stairstep traps, it underwent primary trapping, with bottom sections achieving 40–50% higher trapping rates than top sections, and a minimal amount was leaked into the top section of the domain. This provides insight into the CO_2_ migration and CO_2_ leakage in a domain embedded with geological features along with geological structures, where pressure variations of up to 10% from initial conditions were observed. At the same time, the temperature remained stable within ± 1 °C of the initial 75 °C throughout the simulation period of nearly 3000 years.

The CO₂ mass flux analysis across the three geological scenarios demonstrates significant variations in CO₂ leakage behavior. In the synthetic domain-1 scenario, the CO₂ mass flux was highest, with an initial value of 32,088 kg/year at year 30, decreasing to 583 kg/year by year 3000. This high flux is attributed to the constrained movement of CO₂ towards the anticline dome, where fractures allowed greater leakage. The synthetic domain-2 scenario, with no structural barriers, showed a more diffusive leakage pattern, starting at 23,374 kg/year at year 30 and decreasing to 600 kg/year at year 3000. This scenario reflects a more gradual spread of CO₂ in all lateral directions. In contrast, the Synthetic domain-3 scenario, characterized by stair-step topography, displayed moderate leakage, starting at 12,000 kg/year at year 30 and reaching 850 kg/year by year 3000, with CO₂ primarily moving laterally. These findings indicate that the presence of geological features such as anticlines and uneven topography significantly impacts CO₂ leakage dynamics, with the anticline scenario showing the highest initial leakage, followed by the synthetic domain-3 and synthetic domain-2 scenarios.

## Data Availability

All data generated or analyzed during this study are included in this published article.
